# 611 Priorities in Global Burns Research Prioritization Setting Partnership: Perspectives from US Burn Patients, Caregivers, Clinicians

**DOI:** 10.1093/jbcr/iraf019.240

**Published:** 2025-04-01

**Authors:** Carisa Parrish, Hollie Richards, Suzannah Kinsella, Robert Staruch, Anni King, Jane Blazeby

**Affiliations:** Children’s Mercy Kansas City; National Institute of Health and Care Research Bristol Biomedical Research Centre; James Lind Alliance, National Institute of Health and Care Research; Oxford University Hospitals; National Institute of Health and Care Research Bristol Biomedical Research Centre; National Institute of Health and Care Research Bristol Biomedical Research Centre

## Abstract

**Introduction:**

Identification of unanswered questions in burn research could help guide targeted efforts to improve knowledge and support consensus in creating clinical guidelines. The current study applied community-based participatory research methods involving burn patients, caregivers, and burn clinicians to provide an approach that may enhance results that are particularly relevant to populations served by this research.

**Methods:**

Using an international recruitment strategy representing 88 countries, a comprehensive survey was administered to adult burn patients, caregivers, and clinicians to explore important aspects of burn care for each group. Qualitative thematic analysis combined with a review of clinical guidelines yielded 52 prioritized domains of burn care. Participants identified their top 10 priorities, which were further ranked during an international consensus meeting using James Lind Alliance methodology. This synchronous international meeting was held online in 2024 with stakeholders from 15 countries, who participated in mixed groups of burn patients, caregivers, and healthcare providers and collaborated to rank-order a final top 10 list of global research priorities in burn care. USA data were extracted for the present study.

**Results:**

Survey 1 was completed by 131 participants in the USA (49 burn patients, 10 caregivers, and 72 clinicians), representing 8% of the total sample (n=1617). Survey 2 was completed by 51 US participants (20 patients, 4 caregivers, 27 clinicians), again representing 8% of total sample (n=630). The Top 3 priority questions based on US data converged with themes identified by the global Top 10 priorities, denoting emphasis on psychological/social aspects of burn care and optimal acute care medical treatments. Further details of study characteristics, top domains, and representative participant quotes will be provided as part of this presentation.

**Conclusions:**

The top 3 priority questions in global burn research identified by US participants as part of an international James Lind Alliance project reflect emphasis on optimizing medical wound care as well as psychosocial considerations for burn patients and caregivers.

**Applicability of Research to Practice:**

The top 10 global priorities as well as the top 3 US priority questions can be used to inform future research to enhance burn care efficacy and patient outcomes. Application of a global perspective may assist clinicians and researchers to collaborate internationally to optimize research and clinical guidelines.

**Funding for the Study:**

National Institute for Health and Care Research (NIHR)

